# Interdisciplinary Tools to Safeguard and Amplify Aquatic Genetic Resource Use: A Foundation for Industrial-Scale Quality Control for Fertilization

**DOI:** 10.3390/ani16020249

**Published:** 2026-01-14

**Authors:** Sarah Bodenstein, E Hu, Zoltan M. Varga, Terrence R. Tiersch

**Affiliations:** 1 Connecticut Sea Grant College Program, University of Connecticut, Storrs, CT 06340, USA; sarah.bodenstein@uconn.edu; 2 Aquatic Germplasm and Genetic Resources Center, Louisiana State University Agricultural Center, 2288 Gourrier Ave, Baton Rouge, LA 70820, USA; 3 Zero Kelvin LLC., Baton Rouge, LA 70816, USA; ehu@0-kelvin.com; 4 Zebrafish International Resource Center, University of Oregon, Eugene, OR 97403, USA; zoltan@zebrafish.org

**Keywords:** aquatic species, fertilization unit, high-throughput cryopreservation, genetic resources management

## Abstract

Genetic resources are becoming increasingly important in aquatic species, especially in sectors such as aquaculture and biomedical research. These advancements, however, lack standardized methodology to consistently improve efficient use of gametes for fertilization and to eliminate male variation during spawning. This study provides a conceptual basis for generalizable quality control in artificial spawning of aquatic species by using interdisciplinary, industrial-scale tools to calculate a fertilization unit (e.g., the amount of sperm required to reliably fertilize the eggs produced by a female). Blue catfish (*Ictalurus furcatus*), zebrafish (*Danio rerio*), and eastern oysters (*Crassostrea virginica*) were used as diverse representative species. Comparisons among aquatic species were reviewed, fertilization units were defined, and a sensitivity analysis was performed to assess how deviations from the fertilization unit could affect artificial spawning efficiency. Overall, reproductive strategy (e.g., gamete biology) and production setting significantly influenced the fertilization unit. Employing a fertilization unit decreased “wasted” sperm and reduced male variability during spawning. Furthermore, fertilization efficiency dropped significantly when sperm use strayed from the fertilization unit, declining with both underuse and overuse, especially in oysters and catfish. Standardizing gamete use in aquatic species is essential for economic planning and achieving commercial-scale production, especially when investing in selectively bred or cryopreserved sperm.

## Simple Summary

Standardized management of the physical genetic material (i.e., sperm or egg cells) of aquatic species is becoming increasingly important for industries such as aquaculture and biomedical research. These industries often develop animals with valuable traits through selective breeding, and protecting these gains requires careful management of genetic material. This study provides a quality control framework for using a consistent amount of sperm when spawning to increase the consistency and efficiency of breeding efforts for aquatic species. Blue catfish, zebrafish, and eastern oysters were used as example species. The quality control framework provided in this study included a formula to calculate the “fertilization unit,” or the amount of sperm required to reliably fertilize the eggs produced by a female. We concluded that using the fertilization unit decreased “wasted” sperm and reduced variability during spawning. Adopting this kind of quality control framework will support more efficient breeding, better business planning, and improved use of valuable genetic resources in aquaculture and aquatic industries.

## 1. Introduction

Artificial fertilization (AF) has become central to many aquaculture and fish production industries, including blue catfish [1], zebrafish [2], and Eastern oysters [3]. Despite this, many hatchery manuals and production guidelines do not specify a quantity of sperm that should be used to fertilize a specific number of females or eggs. Guidelines often neglect sperm concentration or density, or offer qualitative assessments such as adjusting concentration based on perceived opacity [3]. Other recommendations include a volume or mass of sperm or testes to be applied per volume of eggs or per number of females [4]. These methods assume sperm are always in abundance and, therefore, can be applied in excess without routine attention to quality management procedures [5]. This has led to inefficiencies and inconsistencies when inducing artificial spawning [2,4] that undermine substantial investments made in selective breeding, artificial fertilization (AF), and cryopreservation. Integrating sperm quality control metrics during artificial insemination is key to managing, optimizing, and improving aquatic genetic resources and production capacity and has been a mainstay for livestock semen industries.

In the United States, freshwater and marine aquaculture production was valued at $1.7 billion in 2022 [6]. This is less than 2% of the total value of another key protein-producing sector, the beef cattle industry. In 2022, the retail equivalent value of beef produced in the United States was over $143 billion [7]. The massive scales that the beef and dairy cattle industries have achieved are due in part to the widespread implementation of high-throughput, efficiency-driven genetic management systems. The livestock industry, especially cattle, embraced selective breeding programs and used managed germplasm to ensure rapid genetic advancement. Cryopreservation and artificial insemination research in farm animals began in Russia in 1899 and reached commercial scale by the 1930s [8]. By the 1950s, inseminations using selectively bred cryopreserved bull semen had become reliable, and the global market followed quickly [8]. Today, Certified Semen Services, Inc. (NAAB-CSS), a subsidiary of the National Association of Animal Breeders, provides international quality standards for semen handling and distributes samples with an array of commercially valuable genetic traits. United States dairy semen exports alone were valued at over $31 million in 2022 [9].

The success of these large-scale industries can be attributed in large part to strict quality control (QC) achieved through the use of interdisciplinary tools. These tools span multiple disciplines, including animal husbandry, cryobiology, industrial engineering, mathematics, and economics [10,11]. Standardization tools like “Services per Conception” ensure semen samples are used effectively and have created the economic market backbone for cattle genetics. This metric expresses the relationship between insemination and pregnancy. Calculating and monitoring Services per Conception requires integration of reproductive biology, husbandry, cryobiology, industrial engineering, and economic analysis. Implementing this metric has enabled precise economic planning in cattle industries and the emergence of a scalable market for genetic products (i.e., semen frozen in 0.5 mL straws) [12]. Fertilization units, such as Services per Conception, are central to what can be called “sperm economics”, the logic and discipline of using sperm efficiently, especially as cryopreserved sperm becomes more widely available and valuable. Indeed, the limiting factor to successful conception is generally recognized as resulting from inadequate classification of female reproductive readiness, rather than problems with the semen dose or insemination procedures [13].

In contrast, aquaculture has a longer documented history of artificial spawning than livestock, but has not yet achieved comparable standardization or commercial efficiency. Artificial spawning was documented in 1758 for trout and salmon and in 1860 for carp [14,15,16]. The first report of cryopreserved fish sperm appeared in 1953 [17], around the same time as in cattle, but aquatic cryopreservation has not progressed to commercial adoption. Currently, most efforts with aquatic genetic resources remain at the research scale, with limited application beyond specific laboratories or farms [18,19]. A particular research facility or farm may be able to freeze sperm and fertilize a predicted number of eggs, but this technology cannot be routinely translated to other facilities or applied at higher production scales. Production scales must range from protocol development at the laboratory scale to high-throughput, commercial production that meets industry demands and attains economic efficiency at the industrial scale [20]. For aquaculture to reach similar potential as the cattle industry, it must shift from volume-based thinking to unit-based thinking and implement sperm quality control. Interdisciplinary approaches must be employed to standardize a fertilization unit concept for aquatic species that can be applied across multiple production scales and species while ensuring consistent fertilization rates. Industrial-scale application of a fertilization unit would support quality control, reduce male variability, and enable back-calculation of broodstock requirements, supporting business modeling and operational planning.

The conceptualization of standardized, interdisciplinary genetic management techniques from livestock has begun in aquatic species such as catfish (*Ictalurus furcatus* and *I. punctatus*), zebrafish *(Danio rerio*), and oysters (*Crassostrea virginica*). These diverse species represent a wide range of production systems, from biomedical research to commercial food production. Research investments to ensure fertilization efficacy are underway, including using ultrasound and machine learning to assess gonad development [21], conducting economic analyses of cryopreservation integration [22,23], and developing high-throughput freezing methods [24,25]. Yet a foundational tool is missing: a standardized method to ensure efficient and effective gamete use [26]. Here, we propose the fertilization unit concept based on practical metrics, such as the amount of sperm needed to reliably fertilize the eggs of a single female, assuming she is in good reproductive condition.

As such, the goal of this study was to define and establish the fertilization unit concept for use as a standardized tool for spawning across aquatic species to improve efficiency, enable economic planning, and support scalable breeding programs. With blue catfish, zebrafish, and eastern oysters as representative species, our objectives were to: (1) cross reference the processes of artificial insemination in cattle and artificial spawning in aquatic species to help define and adapt the components of a fertilization unit; (2) calculate fertilization units using the components of hatchery production in three aquatic species; (3) evaluate the required quantity of broodstock and genetic inputs based on production scales of aquatic species, and (4) develop an economic analysis for implementing the fertilization unit in a hatchery or laboratory setting. This approach will define a genetic product—a unit of sperm capable of reliably fertilizing a set number of eggs—that can provide a basis for back-calculations to original broodstock numbers and projections into cost analyses and business planning. Importantly, it would also lay a foundation for the development of markets based on the distribution of genetic improvement in the form of frozen sperm (e.g., instead of reliance on live animals). It is important to recognize that this approach does not exist in current practices across aquaculture, despite centuries of hatchery work.

## 2. Materials and Methods

### 2.1. Components of the Fertilization Unit

To begin defining the components of a fertilization unit for aquatic species, comparisons were drawn from an existing industrial system used for genetic resource exchange: the global bull semen industry. To gather information on how semen is preserved and used in the cattle industry, trips were made to central sperm cryopreservation facilities (i.e., “bull studs”) beginning in 2008 to observe the process of semen cryopreservation and fertilization unit calculation. In addition, discussions with technicians provided vital information regarding industrial cattle genetic improvement. A literature review of techniques and applications of cryopreserved cattle sperm was also conducted to gather in-depth analysis [27,28,29]. This work outlined the basic components of fertilization unit calculations. In addition, relationships and equivalencies were charted to align life history and husbandry steps within the bull industry to practices used for catfish, zebrafish, and oysters. These three species were selected to represent a wide range of life history attributes (e.g., reproductive biology, body size, survival rate), environmental conditions, and production systems in aquatic species.

To apply fertilization unit calculations to the three aquatic organisms in this study, information was gathered about life-history stages and breeding techniques. For catfish, research focused on the production of hybrid catfish (commonly used within the industry), which are bred by crossing male blue catfish (*I. furcatus*) with female channel catfish (*I. punctatus*). Research trips included visits to the USDA-ARS Warmwater Aquaculture Research Unit (WARU, Stoneville, MS) and hybrid catfish hatcheries in AR and MS. During these trips and collaborations since 2008, all production data (e.g., male quality, sample QC, process records) were recorded during high-throughput processing, and all hatchery data (e.g., egg quality, sperm usage, neurulation rate, hatching rate) were tracked during application [30].

To apply fertilization unit calculations to zebrafish (the leading aquatic biomedical research model), in-depth discussions and collaborations with personnel at the Zebrafish International Resource Center (ZIRC) were used to understand cryopreservation, husbandry, and dissemination processes (Joy M. Murphy, pers. comm., Tech. Assistant at ZIRC, University of Oregon). These collaborations have taken place continuously since 2006. Currently, ZIRC has cryopreserved over 46,000 distinct genetic modifications in lines of zebrafish (zfin.org, accessed on 8 October 2025) to reduce the economic burden of live animal maintenance, as well as to reliably reconstitute and disseminate specific alleles to research communities. Through discussions and production analysis, relevant data such as sperm concentration, sperm volume, and number of fish processed were collected.

Finally, to apply fertilization unit calculations to oysters, previous research and author experience since 1995 were used to understand commercial production and the cryopreservation processes. Previous research included studies that pioneered Eastern and Pacific oyster cryopreservation methods [27,31,32], as well as work that investigated commercial applications of oyster cryopreservation [33,34]. Additional insight was provided by visits to and discussions with staff at the Louisiana Sea Grant Oyster Lab and the Michael C. Voisin Oyster Hatchery on Grand Isle, LA, as well as the Auburn University Shellfish Lab. Through experience, previous production analysis, and hatchery visits, relevant data such as sperm and egg volumes, fertilization and survival rates, and sperm concentration were collected. In addition to on-site visits and discussions for each aquatic species, literature reviews of catfish, zebrafish, and oyster production were conducted as needed.

### 2.2. Fertilization Unit Calculations

After the components of stock center or hatchery production (i.e., key life stages, fertilization and survival rates) were defined for each aquatic organism, they were applied to calculate a fertilization unit (Equations (1) and (2)). It is important to note that while the fertilization unit refers to the number of sperm needed to fertilize all the eggs from a single female, one “unit” could contain multiple containers of sperm, such as multiple French straws. To begin the calculation for fresh sperm, the average number of viable eggs produced by a single female (No. of Eggs) is multiplied by the ratio of the number of sperm necessary to fertilize each egg (*Sperm:Egg Ratio*). The resulting value, the quantity of required sperm, is divided by the concentration produced by dilution upon collection from the aquatic species, and this calculated volume (in mL) represents the fertilization unit (Equation (1)).

(1) $$\underset{\left(\mathrm{fresh sperm}\right)}{\mathrm{Fertilization Unit}}\;=\frac{No.\;viable\;Eggs\times Sperm:Egg\;Ratio}{Sperm\;Concentration}$$

The beginning of the equation remains unchanged when calculating the fertilization unit for cryopreserved sperm. The No. of Eggs and *Sperm:Egg Ratio* are multiplied to calculate the quantity of required sperm. The quantity of sperm is divided by the sperm concentration within a cryopreservation container (i.e., a French straw, IMV Technologies Cat#: 005584, IMV Technologies, Normandy, France, or Cryo-vial, Thermo Scientific Cat#: 12-570-201, Thermo Fisher Scientific Inc., Waltham, MA, USA) to calculate the necessary volume of sperm. The necessary volume of sperm is divided by the volume of the cryopreserved material per freezing container (*Vol. Container*) to calculate the number of containers needed to fertilize all eggs produced by a single female or the fertilization unit (Equation (2)). When the fertilization unit is calculated for cryopreserved sperm, the number of freezing containers should be rounded up to the nearest whole number to avoid partial use of a container.

(2) $$\underset{\left(\mathrm{cryopreserved sperm}\right)}{\mathrm{Fertilization Unit}}\;=\frac{No.\;viable\;Eggs\times Sperm:Egg\;Ratio}{Sperm\;Concentration}\;÷Vol.\;Container$$

### 2.3. Production and Sperm Requirements

In addition to the fertilization unit, the total quantity of sperm required for production (at scale) of the three aquatic species was calculated. These calculations were based on the use of cryopreserved sperm. For each species, a target number of offspring produced from a single spawn was chosen based on real-world values. Fertilization and survival rates of embryos or larvae were combined with sperm concentrations to estimate the amount of sperm required to fertilize a quantity of eggs and ensure a certain number of offspring survived to adulthood. Finally, the number of sperm required, and the volume of the freezing containers were considered to calculate the number of containers (e.g., French straws or cryo-vials) needed to produce the target number of offspring.

For catfish, the sperm requirements were calculated during visits to the USDA-ARS WARU. Neurulated embryos (or Stage V embryos) of hybrid catfish were evaluated at 27–30 h after fertilization at 26–28 °C by viewing with the naked eye and back illumination [35]. Fertilization rate was expressed as the percentage of neurulated embryos in relation to the total number of eggs used for fertilization. The survival rate was calculated by counting the number of “swim-up fry” at 10 d after fertilization, a juvenile life stage after the fish has left the chorionic (egg) membrane and begun active feeding [28]. The ratio between swim-up fry number and the total number of eggs (estimated volumetrically) was termed the “Swim-up Fry Survival Rate” and was used to express survival rate. This approach was based on commercial hatchery practices for estimating spawning success. In commercial settings, it was assumed that only high-quality broodstock (based on visual indicators or age) are selected for spawning, and therefore broodstock age would not affect spawning success [36]. This assumption was also applied to zebrafish and oyster spawning practices [3,37]. The sperm concentration and number of eggs produced per female were also recorded during these visits.

For zebrafish, the average number of eggs produced per female, sperm-to-egg ratio, sperm concentration, and fertilization and survival rates were obtained through discussion with experienced staff at ZIRC and through literature review [29,38,39]. The fertilization rate was based on the number of unfertilized eggs and developing embryos at 3 h post fertilization (hpf). The survival rate was based on the number of offspring surviving to the target life stage of 24, 48, and 120 hpf, and 28 days post fertilization (dpf) divided by the number of fertilized eggs. This approach was based on standard practices used at the ZIRC and within the zebrafish research community.

Similarly, for oysters, discussion with hatchery managers, author experience, and published data were used to obtain average sperm concentrations, fertilization, and survival rates [27,31,33,34]. The average number of eggs produced per female, sperm-to-egg ratio, and average fertilization rate were used to estimate the number of larvae at 24 h after fertilization. It should be noted that while the percent sperm viability data were not collected, fertilization rate data for each species implicitly incorporated sperm viability differences between samples. Average survival rates at each subsequent life stage were used to estimate the number of oysters surviving to the “spat” stage (1–2 months old, ~2–4 mm in length) [33]. This approach was based on commercial hatchery practices for estimating spawning success. After these husbandry metrics (components of hatchery production) were obtained for each species, the following equations (Equations (3)–(5)) were used to calculate the number of freezing containers needed to produce a desired number of offspring:

(3) $$No.\;viable\;Eggs=\frac{No.\;Offspring}{Survival\;Rate}÷Fertilization\;Rate$$

where *No. viable Eggs* is the number of eggs required to produce a desired number of offspring (*No. Offspring*) based on the survival and fertilization rates.

(4) $$Vol.\;Sperm=\frac{No.\;Viable\;Eggs\times Sperm:Egg\;Ratio}{{Sperm\;Concentration}_{container}}$$

The volume of sperm required (*Vol. Sperm*) to fertilize the No. Eggs was calculated using the sperm-to-egg ratio (*Sperm:Egg Ratio*) and the average sperm concentration in a freezing container (*Sperm Concentration_container_*).

(5) $$Required\;Sperm\;Containers=\frac{Vol.\;Sperm}{Vol.\;Container}$$

Finally, the number of freezing containers (*Required Sperm Containers*) required to hold the required volume of sperm was calculated based on the container volume (*Vol. Container*).

### 2.4. Sensitivity Analysis of the Fertilization Unit

Adoption of cryopreserved products in hatchery or stock center management can provide many advantages if quality management practices are in place to ensure consistency. However, using sperm for production without implementing fertilization units leads to inefficiencies and higher costs. Simply applying fertilization unit calculations will improve efficiency because quantification becomes part of the procedure. To evaluate utility within application of the fertilization unit, a sensitivity analysis was conducted to assess how even small deviations (which would be essentially invisible compared to existing methods) would affect fertilization efficiency. In this case, efficiency was defined as the fertilization output normalized by the cost of germplasm. First, an equation to estimate fertilization efficiency using the fertilization unit was constructed in Equation (6):

(6) $$UFE=\frac{FE}{\left(Ps\times S)+(Pe\times E\right)}$$

where UFE is the Unit Fertilization Efficiency, FE is the estimated target number of fertilized eggs, Ps is the price of sperm, S is the quantity (number) of sperm used in fertilization, Pe is the price of eggs, and E is the quantity (number) of eggs used in fertilization.

Next, equations were developed to estimate fertilization efficiency under two scenarios: overuse (*k* > 1), where sperm was used in excess of the fertilization unit and fertilization rates reached a maximum threshold; and underuse (*k* < 1), where less sperm than the fertilization unit was used resulting in a linear decline in fertilized eggs (Equations (7) and (8)).

(7) $${UFE}_{k>1}=\frac{FE}{\left(Ps\times k\times S)+(Pe\times E\right)}$$

(8) $${UFE}_{k<1}=\frac{k\times FE}{(Ps\times k\times S)+Pe\times E}$$

where k is the deviation from the fertilization unit. For example, using half of the recommended fertilization unit would result in k = 0.5. The effect of deviating from the fertilization unit was assessed by calculating the percent change of UFE to ${UFE}_{k}$ (Equation (9)). The brackets denote absolute value. Full methodology for simplifying the equations can be found in the Supplementary Material.

(9) $$∆UFE\%=\left|\frac{UFE-{UFE}_{k}}{UFE}\right|\times 100\%$$

## 3. Results

### 3.1. Components of the Fertilization Unit

**Figure 1:**
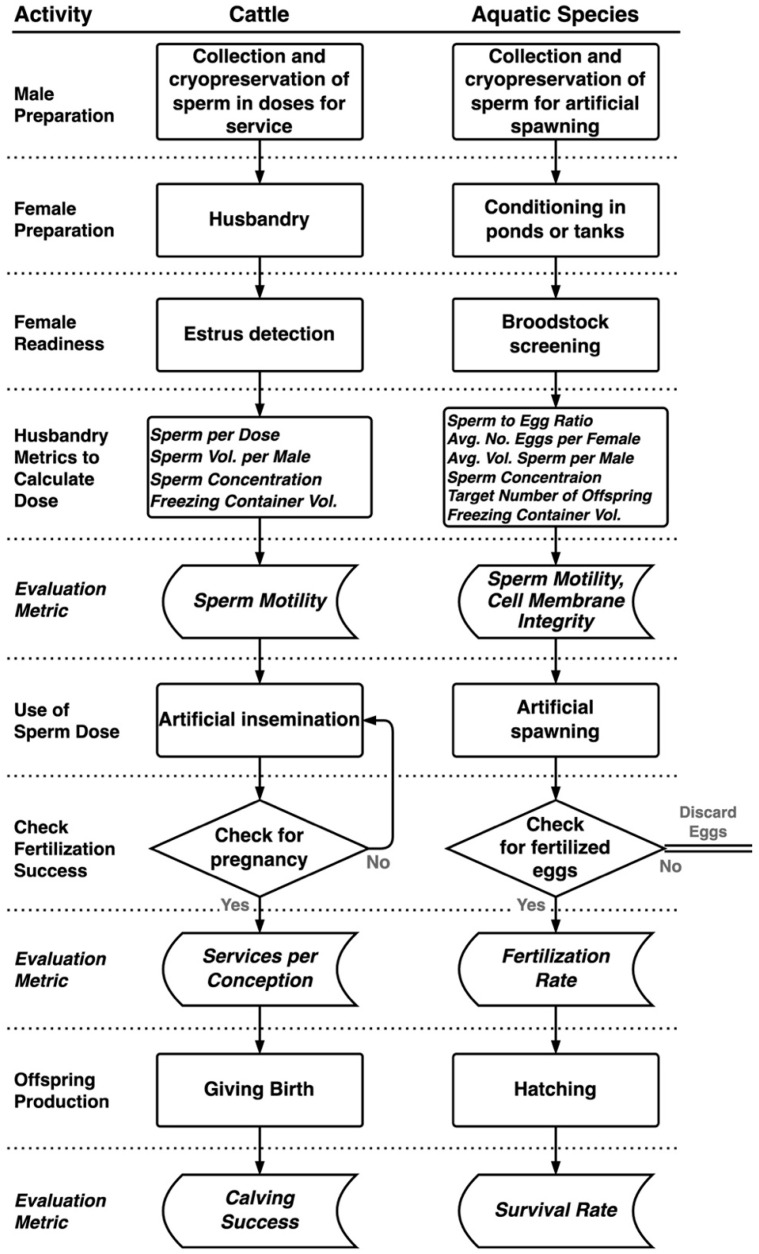
A relational comparison of activities associated with commercial application of artificial insemination in cattle and artificial spawning in three representative aquatic species (catfish, zebrafish, and oysters). The arrows in the charts indicate the sequential flow of processes and the shapes of objects represent different functions: processes (rectangular), decisions (diamond), or data recording (rectangle with two rounded sides). Specific husbandry and evaluation metrics are indicated in italics. Conceptual equivalency between the terrestrial and aquatic reproductive processes is indicated by horizontal alignment of steps.

For comparison purposes, the life history and husbandry techniques for the three species (catfish, zebrafish, and oysters) were aligned with corresponding established activities within the bull semen industry (Figure 1). This comparison aided in identifying the components to calculate a fertilization unit for aquatic species. For cattle, the husbandry metrics to calculate the fertilization unit were Sperm Dose per Egg, Sperm Volume per Male, Sperm Concentration, and Freezing Container Volume [40,41]. For aquatic species, the metrics were similar, although terminology differed slightly. The cattle industry and aquatic species both use motility as a quality-control metric for sperm evaluation. In addition, aquatic researchers sometimes use cell membrane integrity (e.g., dye permeability) as a quality control metric [42,43,44]. The evaluation metrics used in the cattle industry to define the success rate of artificial inseminations were Services per Conception and Calving Success [12]. For the aquatic species, fertilization and survival (at a chosen life stage) rates were identified as roughly equivalent evaluation metrics (Figure 1). One major difference was that aquatic organisms are often r-selected species that produce large quantities of gametes and, consequently, have the potential to generate many offspring at once, unlike cattle. Therefore, additional terminology, such as Target Number of Offspring and Average Number of Eggs per Female, was needed to account for differences in reproductive strategy [24,29,30].

### 3.2. Fertilization Unit Calculations

To calculate the fertilization unit for each species (catfish, zebrafish, and oysters) using cryopreserved sperm, the average values for the required metrics were summarized in Table 1. The average number of eggs (per female), sperm-to-egg ratio, sperm concentration (within one freezing container), and volume of the sample per freezing container were used to calculate the fertilization unit based on Equation (2). To fertilize all eggs produced from one female required 3 French straws for catfish (2700 eggs), 1 Cryo-vial for zebrafish (150 eggs), and 6 straws for oysters (2 × 10^7^ eggs) (Table 1).

### 3.3. Production and Sperm Requirements

The husbandry metrics (components of hatchery production) needed to calculate the sperm requirements for routine production of each species using cryopreserved sperm were summarized in Table 2. A target number of offspring for a single fertilization event for each species was chosen based on average production numbers [30,33]. Target number in combination with fertilization rate, survival rate (at a chosen life stage), sperm-to-egg ratio, sperm concentration (within one freezing container), volume of sample per freezing container, average sperm volume (per male), and average number of eggs (per female) were used to calculate the required sperm based on Equations (3)–(5). Daily catfish production required 791 straws, zebrafish required 1 Cryo-vial, and oysters required 94 straws (Table 3).

### 3.4. Sensitivity Analysis

**Figure 2:**
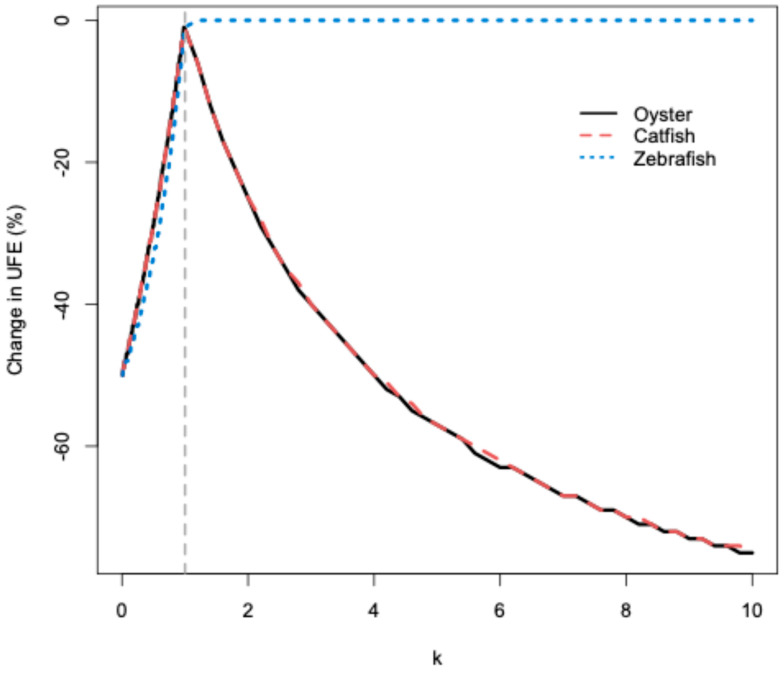
Percent change in fertilization efficiency (UFE) for k = 0.01 to 10 for catfish, zebrafish, and oysters. The vertical gray line indicates k = 1, where there is no deviation from the fertilization unit. All other calculated UFE values (where k ≠ 1) are compared to this value and expressed in percent change in UFE.

As an extremely conservative analysis, the effect on fertilization efficiency of a small deviation from the fertilization unit was assessed, with k ranging from 0.01 to 10 for catfish, zebrafish, and oysters (Figure 2). For k values between 0.01 and 1, the effect on the efficiency (the slope) was similar for catfish and oysters (least squares means comparison, *p* = 0.9). For zebrafish, there was a small but statistically significant difference in slope compared to catfish and oysters (least squares means comparison, *p* ≤ 0.04 for all comparisons). For example, when k = 0.5, catfish and oysters had a decrease in fertilization efficiency of approximately 29%. Zebrafish saw a decrease of 33%, a 4% decrease in efficiency compared to the other two species at the same k value.

For k values between 1 and 10, the effect on the efficiency (the slope) was again similar for catfish and oysters (least squares means comparison, *p* = 0.9). As k increased for these two species, the fertilization efficiency gradually declined (negative logarithmic relationship, *p* ≤ 0.4 for all comparisons, Figure 2). For zebrafish, increasing k also had a negative effect on efficiency. However, the effect was negligible inside the range of k values tested in this study (negative logarithmic relationship, *p* ≤ 0.01, Figure 2). Therefore, the slope for zebrafish differed significantly from those observed in catfish and oysters (least squares means comparison, *p* < 0.01 for all comparisons). For example, when k = 2, zebrafish saw no decrease in fertilization efficiency while catfish and oysters had a decrease of approximately 25%, resulting in a 24% greater decrease in efficiency for catfish and oysters compared to zebrafish at the same k value. As the k values increased, the difference in the effect on efficiency between zebrafish versus catfish and oysters became more pronounced. When k = 5, the decrease in fertilization efficiency for zebrafish remained at less than 1%, while catfish and oysters had a decrease of approximately 57% (a 56% greater decrease). Finally, when k = 10, the decrease in fertilization efficiency for zebrafish remained at less than 1%, while catfish and oysters had a decrease of approximately 75% (a 74% greater decrease).

## 4. Discussion

With the need for increased selective breeding, cryopreservation, and artificial spawning in aquatic species, more efficient use of germplasm is also needed to fully capitalize on emerging opportunities and support large-scale production. For example, gametes can be “wasted” by lack of tight control of numbers and ratios, necessitating the use of excess numbers of broodstock animals to produce additional fertilization attempts [31,48]. If a volume of sperm is mixed with more eggs than recommended for fertilization (i.e., a reduced sperm-to-egg ratio), some potentially fertilizable eggs may not receive sperm, reducing output. Conversely, if an excess of sperm is used (i.e., an increased sperm-to-egg ratio), motile sperm would be wasted. Adoption of recommended fertilization units to ensure quality control would make outcomes more reliable and efficient. This is a fundamental requirement for cryopreserved sperm to become a reliable product form for distribution, exchange, and sale of valuable genetics, and is an absolute requirement for development of industrial standards. This study adopted an interdisciplinary approach, applying animal husbandry and cryobiology research, industrial engineering principles, and theoretical mathematics, to develop a fertilization unit concept for aquatic species with different life histories and diverse economic and scientific uses. Calculations relied on the husbandry metrics of a set average number of eggs (per female), sperm-to-egg ratio, sperm concentration, and volume of the gamete-activating fluid (e.g., tank water) in a container (Equation (2)).

Of these metrics, sperm-to-egg ratio and sperm concentration greatly influenced the fertilization unit. If either value was increased or decreased by one order of magnitude, the fertilization unit changed correspondingly. For example, in catfish, if the sperm concentration was adjusted to 1.0 × 10^8^ cells mL^−1^ (instead of 1.0 × 10^9^ cells mL^−1^) the resulting fertilization unit would be 30 straws per female instead of 3. It is extremely important to note that current hatchery practices would typically not be able to differentiate between these concentrations. In addition, at small scales of production, this difference in the required amount of genetic material may be negligible, for example, in benchtop research studies. At larger industrial scales, however, this difference can render production uneconomical and unsustainable. For example, if one million hybrid catfish fry must be produced daily at a hatchery during the spawning season (a reasonable industrial production scale, [49], appropriate sperm concentrations could be the difference between purchasing 791 straws (Table 3) or almost 8000 straws. This example exhibits the importance of adjusting sperm concentration to relevant use [50,51,52,53], and ensuring that sperm collection and storage methods are scalable, particularly in high-throughput production systems [33,35]. Commercial hatchery protocols often lack methodology to measure sperm concentration [3,36]. Recent studies, however, have detailed scalable methods for measuring and adjusting concentration using a spectrophotometer or microscope counting slides, and buffer solutions [46,50,51,52,53]. For sperm metrics to be practical in artificial spawning, collection protocols should establish targets for minimum fertilization thresholds and consistent results instead of maximizing fertilization numbers often with diminishing economic returns [48], which is frequently pursued in research settings. Future research should establish taxa-specific methodology for recording these sperm metrics, leveraging interdisciplinary approaches such as process mapping [54] to organize and describe protocol steps, and simulation modeling [33,55] to evaluate the time, labor, and equipment requirements, thereby ensuring scalability and standardization.

When calculating a fertilization unit and required sperm metrics for production for any aquatic species, the life history of the animal and characteristics of the user community must be considered carefully and balanced. For cattle, only one offspring is expected to be produced per female, and calves are raised for 1–2 years before moving on to the next production stage [56]. For an aquaculture species such as catfish or oysters, females produce thousands to millions of eggs. Hatcheries are expected to produce millions of juveniles each year for farmers or for stocking recreational populations, which will be ready for farm grow-out within months of spawning [3,30]. Therefore, the sperm requirements for production aquaculture settings can be substantially different from requirements for cattle production or warehousing of biomedical models like zebrafish, which produce only few hundred eggs (Table 3).

On average, zebrafish females produce only around 200 eggs per week and production demands require an average of 100 embryos be produced per resource center user request. Thus, females and eggs constitute an operational bottleneck. However, these relatively lower gamete quantities suit the production model of biomedical stock centers, where spawns are driven by individual user requests for starting their own research populations [57]. Consequently, the cryopreservation protocol for zebrafish sperm allows for one cryo-vial to contain more than the total amount of sperm required for one fertilization unit and guarantees fulfilling production requirements at the first attempt (Table 1 and Table 3). This practice optimizes fertilization by focusing on the primary limiting factor: the availability and quality of eggs. Repeated fertilization attempts, and the associated stress on females, are avoided, and the need to reschedule thaws, IVF procedures, and shipments is reduced. Therefore, laboratory animal welfare is enhanced, while operational costs and effort are minimized. By calculating the fertilization unit and evaluating it in the context of life history, production systems, and user needs, necessary improvements can be made to the efficiency and consistency of sperm collection, storage methods, and production requirements. This translational approach is essential when scaling from research studies to community-level or industrial-scale application and was a required step to develop the global markets that exist for bull semen. Successful implementation also requires a variety of interdisciplinary tools. These include animal husbandry research to assess life histories [3,39,47], industrial engineering tools such as process mapping [54] and simulation modeling [55] to evaluate production systems, and economic tools such as partial budgeting to ensure cost-effectiveness [58].

Through these interdisciplinary tools, implementing fertilization units in artificial spawning provides an opportunity to standardize fertilization success and increase consistency by excluding sperm or male variability from fertilization outcomes. Many conditions, such as facility architecture and infrastructure, source water availability and quality, and food quality, are difficult to standardize among hatcheries or spawning facilities due to geographic or management reasons. In general, fertilization and survival rates are a function of male variation, female variation, and facility variation (i.e., variation in water quality) [59,60,61]. By use of standardized fertilization units, male variation can be controlled or eliminated. To ensure consistency of fertilization units, two criteria must be met. The first is that the units of sperm (cryopreserved or fresh) must be produced within species-specific or operation-specific quality control metrics. Such metrics evaluate material (i.e., sperm) throughout the production process and identify and eliminate deficiencies and defects [62].

The percentage of motile sperm (sperm motility) is a common quality control metric used in the dairy industry [63], and a quality evaluation method in catfish, zebrafish, and oyster cryopreservation research [64,65,66]. It is important to note that quality control (QC) is an industrial practice used to remove defective materials during a process (i.e., product-based disposal based on set thresholds) [67], and quality evaluation is a research tool used to collect data for analysis. Thus, by evaluating sperm motility for use in QC, poor-quality samples would be identified and discarded early (e.g., before freezing, thereby avoiding wasted effort), ensuring that the quality of sperm used in fertilization is consistent. While sperm motility may not be directly correlated with fertilization rate [32], if all samples are held within a specified motility range (e.g., indicating good overall cellular integrity), consistent fertilization results can be obtained. Individual facilities can define their own thresholds for what constitutes a poor-quality sperm sample. Having a quantitative measurement of sperm quality associated with each sample is the key component for consistent fertilization success [13,19]. Furthermore, if sperm quality was evaluated, the number of females needed to provide eggs for sperm quality control purposes could be minimized or entirely circumvented, improving animal welfare.

The second criterion is that the sperm must be used in fertilization following standardized protocols, including correct use of the fertilization unit. This criterion ensures that the number of eggs fertilized by one straw is functionally within the fertilization unit. In addition, during spawning, facilities must employ proper egg collection techniques and provide a suitable spawning environment. All these factors eliminate as much variation as possible during spawning and increase the reliability of offspring production. While much of male variability is removed by using quality-controlled fertilization units, female and facility variability remain a challenge [48,61,68]. A practical method, practiced by ZIRC, that hatcheries or spawning facilities could employ to reduce this variability is keeping records tracking identifiable quality factors and fertilization outcomes. For example, most hatcheries and biomedical fish facilities keep records of water quality, broodstock provenance, and physiological characteristics of broodstock during conditioning [3,69]. With a fertilization unit approach, hatcheries could quantitatively evaluate fertilization and survival success against these factors and improve production protocols accordingly.

Although some factors cannot be controlled, such as source water quality in flow-through systems, hatcheries can plan for reduced survival and adjust fertilization units and production schedules. Beyond the hatchery stage, the effect of facility conditions and husbandry practices on adult well-being and survival has been poorly studied outside the biomedical field. Even if variation is reduced, the hatchery exposes animals to some degree of stress. Differential survival in juveniles may occur due to these stressors, resulting in selection events that affect adult survival or growth [70,71]. Keeping records of hatchery conditions (i.e., water quality and husbandry protocols) helps explain and plan accordingly for variabilities in adult performance.

Among the controllable factors in hatchery operations, the amount of sperm used during artificial fertilization plays a key role in fertilization success [50,51,52] and efficiency. The extremely conservative sensitivity analysis used in this study indicated that using half the recommended fertilization unit (k = 0.5) led to an approximate 30% decline in fertilization efficiency across all three species. This decrease was slightly more pronounced in zebrafish, indicating that the artificial spawning practices used for this species were more sensitive to insufficient sperm numbers. Nonetheless, across all species examined, insufficient sperm numbers had a negative effect on fertilization efficiency, highlighting the importance of properly applying the fertilization unit during artificial spawning to ensure reliable production. As indicated above, these small differences in sperm numbers would not be detectable with standard hatchery practices, and the range in sperm numbers used in research replicates is typically not estimated or controlled [3,49].

Overall, zebrafish were least sensitive to decreases in fertilization efficiency due to overuse of sperm (k > 1). As the overuse of sperm increased, oysters and catfish saw increasingly more pronounced decreases in efficiency compared to zebrafish. This difference in sensitivity to sperm overuse was likely due to the relatively low target number of fertilized eggs used for zebrafish (150), compared to 1.1 × 10^4^ eggs for catfish and 2 × 10^7^ eggs for oysters. However, in all three species, the sperm concentration was at least 1 × 10^8^ sperm per milliliter. Zebrafish were less sensitive to the overuse of sperm because the fertilization protocol (based on the standard protocol used at ZIRC) and the resulting fertilization unit already included the use of excess sperm. It is standard practice to use more than enough sperm (66 times more than necessary) to fertilize eggs from a single female zebrafish [29,38,39].

While ZIRC could afford to reduce sperm concentration without impacting production requirements and freezer space efficiency, variations in egg quality and quantity could reduce fertilization success, especially in mutant or inbred lines with reduced fecundity. Sudden changes in the numbers of customer orders, and workforce turnover can also affect timing in the methods associated with thawing and use. These problems are inherent to serving as a national stock center, and would necessitate multiple fertilization attempts, delay fulfillment of user requests, increase workhours (i.e., personnel cost) per request, and create scheduling problems. Because their business model is based on timely fulfillment of customer orders, it is advantageous to include a safety factor in sperm numbers to offset potential problems. However, because the fertilization unit is controllable, it can be adjusted in the future to satisfy changing needs.

ZIRC provides a genetic inventory of over 46,800 distinct genetic modifications that can be used in research (zfin.org). Customers pay a much higher price per fish ($400 per transgenic adult pair, or 100 offspring from an identified pair; https://zebrafish.org/documents/fees.php, accessed on 8 October 2025) than customers buying most commercial aquaculture products, which do not need molecular genetic identification. To cope with static budgets, ZIRC has used cryopreservation to greatly reduce the costs associated with preserving lines by transitioning from continuous maintenance of live populations to frozen storage of sperm. Zebrafish stock centers use frozen sperm that contain specific alleles (genetic modifications) to fertilize eggs, and this sperm is often available in limited quantities (collections of 1–5 µL per male of undiluted sperm). Despite this, intentionally over-using sperm ensures that each user request has virtually 100% success rate on the first attempt, and ensures a tight turnaround time at ZIRC, with minimal time, effort, and materials used. The fertilization unit as applied at ZIRC could be used to improve the operational efficiency of zebrafish distribution. A greater quantity of eggs could be fertilized with the same amount of sperm, or a slightly lower sperm concentration could be used to fertilize the same number of eggs. These modifications would extend the time needed between reamplifications of cryopreserved lines because more samples could be preserved from the same number of males.

In commercial aquaculture species such as catfish and oysters, overuse or underuse of sperm resulted in notable decreases in fertilization efficiency. If farmers invest in cryopreserved germplasm with selectively bred traits without using the fertilization unit, the return on investment would be reduced, especially with increases in scale of production. In swine, the efficiency and consistency of artificial insemination is a leading factor in the profitability of farm operations [72]. In the cattle industry, even considering the costs of genotyping and selective breeding methods, selectively bred germplasm used in combination with insemination offers double the economic advantage over a traditional program [73]. As stated above, these industries have standardized sperm use for insemination, such as Services per Conception in cattle [12]. In aquaculture, sperm is routinely wasted during artificial spawning either through overuse, which provides no additional fertilization beyond what the fertilization unit would achieve, or underuse, which results in lower fertilization success. The sensitivity analysis clearly demonstrated significant decreases in efficiency if sperm application deviated slightly from the fertilization unit during artificial spawning. Therefore, implementing a fertilization unit in aquaculture is necessary for hatcheries to efficiently use cryopreserved sperm and see a positive return on investment. Most importantly, industry standards can be based on the fertilization unit approach and lay the foundation for the development of dependable markets for improved germplasm that can be sold in reliable and easily transportable product forms (e.g., straws vs. live animals).

It should be noted that the equations developed for the sensitivity analysis may not perfectly capture changes in fertilization efficiency for certain species or applications. As is the case with zebrafish, the number of eggs produced by a female and subsequently fertilized is substantially smaller, approximately six orders of magnitude lower, than the number of sperm used. Due to the assumptions in the equations regarding a linear relationship between sperm and egg numbers and fertilization success, the calculated results may not fully reflect real-world efficiency changes. Additionally, these assumptions may more accurately reflect the sensitivity of commercial-scale hatchery production, where cost-efficient fertilization is imperative, rather than the line recovery business model used in biomedical research. In future studies the sensitivity analysis equations could be modified for species with different life history traits and commercial applications. This could include commercial hatchery species like catfish and oysters, which are produced in large numbers with sperm and egg counts differing by only a few orders of magnitude; biomedical research species like zebrafish, which are produced in smaller numbers and exhibit a greater disparity in gamete numbers; and live-bearing species used in research, such as Xiphophorus.

## 5. Conclusions

Gaining the benefits of quality control through interdisciplinary concepts, such as the fertilization unit, is necessary to scale production capacity in aquatic systems. This concept signals a paradigm shift, from volume-based thinking, where sperm are always in abundance, to unit-based thinking, where sperm are a resource that must be carefully managed. Implementing a fertilization unit not only enables more consistent and predictable fertilization outcomes but also supports economic planning and promotes efficient use of sperm. Economic planning is especially critical when using high-value, selectively bred genetic resources, particularly in industrial-scale operations, where initial investments in genetic material are substantial [72]. Standardizing the fertilization unit across aquatic species also enables downstream benefits such as repository development, genetic traceability, and intellectual property protection. A standardized metric could underpin germplasm certification, support repository-based quality assurance, and establish transparent frameworks for the exchange and commercialization of genetic resources. In essence, it transforms cryopreservation from a laboratory technique into an operational and regulatory foundation for aquatic genetic resource management and commercialization. These concepts are becoming increasingly important as interest in another interdisciplinary tool, cryopreservation, continues to grow in aquatic systems.

Cryopreserved sperm and repository capabilities have the potential to expand the utility and value of aquatic genetic resources, especially through time with the accumulation of associated genetic and production data. In aquaculture, cryopreserved sperm can accelerate selective breeding programs by facilitating germplasm exchanges between hatcheries and safeguarding valuable genetic traits against disease or natural disasters [34,35]. In biomedical research, the numerous genotypes of model organisms can be preserved reliably and economically to reduce the burden of live animal maintenance and increase the ease of dissemination [46,74]. In conservation, programs for aquatic species can cryopreserve sperm from wild populations and develop germplasm repositories for future repopulation of lost genetic and animal diversity [75]. Despite benefits such as these, which have been demonstrated in other commercial-scale industries such as livestock and crops, cryopreservation and repositories have not been brought into most aquatic production systems.

One reason for this is that aquatic cryopreservation research has resided mostly in traditional (reductionist) data-gathering approaches focused on the protocol level (the research scale), whereas application would be focused on the higher functional level of germplasm repository activities (the product level and industrial scale). Research has attempted to optimize specific values at specific steps, such as sperm motility. Application would seek to integrate and balance results across all steps to yield a reliable, economical, functioning overall process. In addition, research tends to be narrow in providing improvements in single factors (e.g., choice of cryoprotective agent or fertilization percent), while application would be more comprehensive in the scope of factors that need to be addressed (e.g., per-unit costs, efficiency, safety, throughput, ease of training).

Another limitation of reliance on traditional research approaches for industrial application is the focus on novelty, which leads to the mistaken notion that cryopreservation protocols are species-specific and must be developed de novo, often in non-reproducible ways. These new protocols often rely on methods and attributes that are not scalable or easily transferable across facilities. For example, the use of freezing containers that cannot be filled, sealed, and labeled with automated processing equipment defeats subsequent attempts to efficiently increase the scale of a research protocol or transfer the protocol to new facilities. Because of these factors, and the almost exclusive focus on protocol development and non-practical optimization over the past 70 years, cryopreservation research in aquatic species has yielded limited application.

To move forward, interdisciplinary work across a variety of social sectors must focus on factors such as scaling, economics, throughput, and quality management in real-world contexts. Proven concepts such as the fertilization unit can provide a common framework across aquatic repositories to support their development, even though species-specific requirements and institutional goals vary. These and other factors have been harmonized before to support multibillion-dollar global industries, such as those for the buying and selling of cryopreserved semen from superior dairy bulls. Rather than reinventing the wheel, aquatic user communities can learn much from interdisciplinary examination of livestock approaches and existing repositories to provide future models for the commercial-scale development of germplasm repositories in aquaculture [76].

## Figures and Tables

**Table 1:** The husbandry metrics (components of production) for catfish, zebrafish, and oysters required to calculate the volume of sperm and fertilization unit for fresh stripped sperm. Fertilization unit calculations were based on Equation (1) using cryopreserved sperm. To perform the calculations the following metrics were needed: Average Number of Eggs (per female), Sperm-to-Egg Ratio, Sperm Concentration (within freezing container), and Volume of sperm in a Freezing Container.

	Avg. No. of Viable Eggs (per Female)	Sperm-to-Egg Ratio	Sperm Concentration	Freezing Container Volume	Vol. Sperm Required	Fertilization Unit (Frozen Sperm)
**Catfish**	$1.1\times 10^{4}$	$1.35\times 10^{5}$ sperm egg^−1^	$1\times 10^{9}$ sperm mL^−1^	0.5 mL (French straw)	1.5 mL	3 straws
**Zebrafish**	150	$2\times 10^{2}\;$ sperm egg^−1^	$1\times 10^{8}$ sperm mL^−1^	0.02 mL (per Cryo-vial)	0.3 μL	1 vial
**Oysters**	$2\times 10^{7}$	15 sperm egg^−1^	$1\times 10^{8}$ sperm mL^−1^	0.5 mL (French straw)	3 mL	6 straws

Note: although Cryo-vials have a total volume of 0.5 mL, standard procedure at ZIRC requires a maximum of 0.02 mL of sperm be aliquoted into each vial.

**Table 2:** The husbandry metrics (components of production) for catfish, zebrafish, and oysters necessary to calculate the required sperm for. production of each species using thawed cryopreserved sperm. To perform production calculations the following values are listed: Target Number of Offspring, Fertilization Rate, Survival Rate (at a chosen life stage), Sperm-to-Egg Ratio, Sperm Concentration (within freezing container), Volume of sperm in Freezing Container, Average Sperm Volume (per male), and Average Number of Eggs (per female). Sources used to create this table include: [29,30,33,39,45,46,47].

	Target No. of Offspring	Fertilization Rate	Survival Rate & Life Stage	Sperm-to-Egg Ratio	Sperm Concentration (in Container)	Freezing Container Volume	Avg. Sperm Volume (per Male)	Avg. No. of Eggs (per Female)
**Catfish**	$1\;\times \;10^{6}$ (Swim-up fry)	67%	51%—Swim-up fry	$1.35\;\times \;10^{5}$ sperm egg^−1^	$1\;\times \;10^{9}$ sperm mL^−1^	0.5 mL French straw	32.5 mL	$1.1\times 10^{4}$
**Zebrafish**	100 (28 days post fertilization, dpf)	60%	95%—28 dpf	$2\;\times \;10^{2}$ sperm egg^−1^	$1\;\times \;10^{8}$ sperm mL^−1^	0.02 mL per Cryo-vial	1.5 μL	150
**Oysters**	$3.3\;\times \;10^{6}$ (Spat)	20%	5.3%—Post-settlement juvenile (spat)	15 sperm egg^−1^	$1\;\times \;10^{8}$ sperm mL^−1^	0.5 mL French straw	40 mL	$2\times 10^{7}$

Note: although Cryo-vials have a total volume of 0.5 mL, standard procedure at ZIRC requires a maximum of 0.02 mL of sperm be aliquoted into each vial.

**Table 3:** The calculated Number of Eggs, Sperm Volume, Required Sperm (in number of freezing containers) for catfish, zebrafish, and oysters are listed based on Equations (3)–(5) and values reported in Table 2. The minimum number of males and females required to produce the Target Number of Offsprings are also listed. Target Number of Offspring values from Table 1 are listed for clarity. Days post fertilization was abbreviated to “dpf”.

	Target No. of Offspring	Calc. No. of Eggs	Calc. Sperm Volume	Required Sperm (No. of Freezing Containers)	Min. No. of Males	Min. No. of Females
**Catfish**	$1\;\times \;10^{6}$ (Swim-up fry)	$2.9\times 10^{6}$	395 mL	791 French straws (0.5 mL each)	13	267
**Zebrafish**	100 (28 dpf)	176	0.4 μL	1 Cryo-vial (0.02 mL each)	1 *	2
**Oysters**	$3.3\;\times \;10^{6}$ (Spat)	$3.1\times 10^{8}$	47 mL	94 French straws (0.5 mL each)	2	16

Note: although Cryo-vials have a total volume of 0.5 mL, standard procedure at ZIRC requires a maximum of 0.02 mL of sperm be aliquoted into each vial. The “*” symbol indicates that when using Equations (3)–(5), the minimum number of males calculated would be 1. While this is feasible following ZIRC cryopreservation protocols, the standard operating procedure generally requires pooling sperm from at least 10 males when collecting sperm.

## Data Availability

The data used to support the findings of this study are available from the corresponding author upon request.

## Supplementary Materials

The following supporting information can be downloaded at: https://www.mdpi.com/article/10.3390/ani16020249/s1.
