# Supplementary material for: Interdisciplinary Tools to Safeguard and Amplify Aquatic Genetic Resource Use: A Foundation for Industrial-Scale Quality Control for Fertilization
Source: Animals (Basel). 2026 Jan 14;16(2):249. doi: 10.3390/ani16020249 (PMC12837652; doi:10.3390/ani16020249)
Supplement: Supplementary file 1 [file animals-16-00249-s001.zip › animals-4053484-supplementary.pdf]

Supplementary Material

Complete calculations for simplifying the Equation 9:

$$UFE = \frac{FE}{Ps \times S + Pe \times E}$$

UFE: Unit Fertilization Efficiency

FE: Fertilized Eggs

Ps: Price of Sperm

S: Sperm Used in the Fertilization

Pe: Price of Eggs

E: Eggs Used in the Fertilization

$$UFE_k = \begin{cases} \dfrac{FE}{Ps \times (k \times S) + Pe \times E} & k > 1 \\ \dfrac{k \times FE}{Ps \times (k \times S) + Pe \times E} & k < 1 \end{cases}$$

k > 1: overuse sperm, i.e. use more than the dose, yet fertilized eggs cap

k < 1: underuse sperm, i.e. use less than the dose, fertilized eggs drop linearly

$$\Delta UFE\% = \left|\frac{UFE - UFE_k}{UFE}\right| \times 100\%$$

$$= \left|1 - \frac{UFE_k}{UFE}\right| \times 100\%$$

k > 1:

$$= \left|1 - \frac{\frac{FE}{Ps \times (k \times S) + Pe \times E}}{\frac{FE}{Ps \times S + Pe \times E}}\right| \times 100\%$$

$$= \left|1 - \frac{Ps \times S + Pe \times E}{Ps \times (k \times S) + Pe \times E}\right| \times 100\%$$

SER (sperm-to-egg ratio) = S/E

$$= \left|1 - \frac{Ps \times SER \times E + Pe \times E}{Ps \times (k \times SER \times E) + Pe \times E}\right| \times 100\%$$

$$= \left|1 - \frac{Ps \times SER + Pe}{Ps \times (k \times SER) + Pe}\right| \times 100\%$$

$$= \left|\frac{Ps \times (k \times SER) + Pe - Ps \times SER - Pe}{Ps \times (k \times SER) + Pe}\right| \times 100\%$$

$$= \left|\frac{Ps \times (k \times SER) - Ps \times SER}{Ps \times (k \times SER) + Pe}\right| \times 100\%$$

$$= \left|\frac{(Ps \times k - Ps) \times SER}{Ps \times (k \times SER) + Pe}\right| \times 100\%$$

$$= \left|\frac{(Ps \times k - Ps) \times SER}{Ps \times (k \times SER) - Ps \times SER + Pe + Ps \times SER}\right| \times 100\%$$

$$= \left|\frac{1}{1 + \frac{Pe + Ps \times SER}{(Ps \times k - Ps) \times SER}}\right| \times 100\%$$

$$= \left|\frac{1}{1 + \frac{Pe + Ps \times SER}{(k - 1) \times Ps \times SER}}\right| \times 100\%$$

$$= \left|\frac{1}{1 + \frac{Pe}{(k - 1) \times Ps \times SER} + \frac{Ps \times SER}{(k - 1) \times Ps \times SER}}\right| \times 100\%$$

$$= \left|\frac{1}{1 + \frac{1}{(k - 1) \times SER} \times \frac{Pe}{Ps} + \frac{1}{k - 1}}\right| \times 100\%$$

Price of gametes is calculated by the price of broodstock dived by the fecundity, i.e.:
Ps = Pb (price of broodstock)/Fs (male fecundity)
Pe = Pb (price of broodstock)/Fe (female fecundity)
Assuming price of broodstock of males and females are the same.
Thus,

$$\frac{Pe}{Ps} = \frac{\frac{Pb}{Fe}}{\frac{Pb}{Fs}} = \frac{Fs}{Fe}$$

So,

$$\Delta UFE\% = \left|\frac{1}{1 + \frac{1}{(k - 1) \times SER} \times \frac{Fs}{Fe} + \frac{1}{k - 1}}\right| \times 100\%$$

k < 1:

$$= \left|1 - \frac{\frac{k \times FE}{Ps \times (k \times S) + Pe \times E}}{\frac{FE}{Ps \times S + Pe \times E}}\right| \times 100\%$$

$$= \left|1 - \frac{k \times (Ps \times S + Pe \times E)}{Ps \times (k \times S) + Pe \times E}\right| \times 100\%$$

SER (sperm-to-egg ratio) = S/E

$$= \left|1 - \frac{k \times (Ps \times SER + Pe)}{Ps \times (k \times SER) + Pe}\right| \times 100\%$$

$$= \left|1 - \frac{k \times (Ps \times SER + Pe)}{Ps \times (k \times SER) + k \times Pe + Pe - k \times Pe}\right| \times 100\%$$

$$= \left|1 - \frac{1}{\frac{Ps \times (k \times SER) + k \times Pe + Pe - k \times Pe}{k \times (Ps \times SER + Pe)}}\right| \times 100\%$$

$$= \left|1 - \frac{1}{1 + \frac{(1 - k) \times Pe}{k \times (Ps \times SER + Pe)}}\right| \times 100\%$$

$$= \left|1 - \frac{1}{1 + \frac{1 - k}{\frac{k \times (Ps \times SER + Pe)}{Pe}}}\right| \times 100\%$$

$$= \left|1 - \frac{1}{1 + \frac{1 - k}{k \times SER \times \frac{Ps}{Pe} + 1}}\right| \times 100\%$$

because,

$$\frac{Ps}{Pe} = \frac{\frac{Pb}{Fs}}{\frac{Pb}{Fe}} = \frac{Fe}{Fs}$$

thus,

$$\Delta UFE\% = \left| 1 - \frac{1}{1 + \frac{1-k}{k \times SER \times \frac{Fe}{Fs} + 1}} \right| \times 100\%$$

In summary,

$$\Delta UFE\% = \begin{cases} \left| \frac{1}{1 + \frac{1}{(k-1) \times SER} \times \frac{Fs}{Fe} + \frac{1}{k-1}} \right| \times 100\% & k > 1 \\ \left| 1 - \frac{1}{1 - \frac{k-1}{k \times SER \times \frac{Fe}{Fs} + 1}} \right| \times 100\% & k < 1 \end{cases}$$

SER: Sperm-to-egg ratio
Fs: Male fecundity
Fe: Female fecundity
k: ratio of using dose
